# Elastic and magnetic characteristics of nano-spinel ferrite Co_0.5_ Mg_x_Cu_0.5−x_Fe_2_O_4_

**DOI:** 10.1038/s41598-024-74484-4

**Published:** 2024-11-27

**Authors:** F. Fakhry, E. Shaheen, H. El-Dosoky, T. M. Meaz, M. Mubark, R. El-Shater

**Affiliations:** 1 Physics Department, Faculty of Science, Tanta University, Tanta, 31527 Egypt; 2 Chemistry Department, Faculty of Science, Tanta University, Tanta, 31527 Egypt

**Keywords:** Co-precipitation Method, XRD, HRTEM, VSM, Nanoferrite particles, Cation distribution, Materials science, Nanoscience and technology, Physics

## Abstract

Wet-chemical co-precipitation was used to create Co_0.5_Mg_x_Cu_0.5−x_Fe_2_O_4_ nano-ferrites (x = 0.0, 0.2, 0.3, and 0.4). XRD, FT-IR, HRTEM, and EDX analyses were used to confirm each sample’s single-phase spinel cubic crystal structure. The crystallite size was calculated from the XRD data and determined to be between (11.1570 and 16.1457 nm), with a lattice constant between (8.359 to 8.387Å). The two absorption bands found in the FTIR data were utilized to show metal cation and oxygen bond stretching at tetrahedral and octahedral positions, as well as to calculate the elastic moduli. The elemental composition and structural behavior of every sample were examined using FE-SEM and EDS. The magnetic parameters were also estimated based on the VSM data, the contribution of magnetic anisotropy (K), and the magnetic interaction by Neel’s and Y-K-type magnetism modify as the Mg^2+^ ion substitution increases, thus we must consider how this variation in cation distribution affects all of these factors. As per the ferromagnet theory, ions originating from the magnetic tetrahedral A and octahedral B sites engage in super-exchange interactions with one another. Anti-ferromagnetic alignment occurs as a result (M_B_-M_A_). Magnetization occurs as a result.

## Introduction

Spinel ferrite, as an essential member of the ferrite family, has a distinct attractiveness. Spinel ferrites have long captured the interest of researchers due to their remarkable properties, which can be seen in a variety of applications such as data storage devices, microwave absorbing materials, ferrofluid and catalysis, gas sensing materials, anode materials of lithium-ion batteries, magnetic hyperthermia, optical and dielectric materials, fast frequency response in soft magnetic materials and biomedical fields, and so on^1^. In addition to their unique physicochemical features, spinel ferrites offer exceptional chemical stability, variable size and shape, surface active sites, a high specific surface area, excellent electric and magnetic properties, and a wide range of scientific and technological applications^2^. The ferrite’s structure may be divided into three types: hexagonal, garnet, and cubic spinel. Of these, cubic spinel ferrites are more adjustable^3^. Spinel ferrite is a form of ferrite that has a cubic structure with the formula MFe_2_O_4_. Divalent cations occupy tetrahedral A-sites in a typical spinel structure, whereas ferrite ions occupy octahedral B-sites. Magnetic materials derived from spinel ferrites have several uses, including wastewater treatment, sensing, electronics, magnetic storage, and contrast agents in magnetic resonance imaging methods^4^. Different synthesis techniques can produce products with varying qualities and properties, which can occasionally define the nature of the materials as a whole^3^. To synthesize ferrite nanoparticles, many methods such as co-precipitation, sol-gel, auto combustion, microemulsion, hydrothermal, and others have been documented^5^. Among these preparation techniques, Co-precipitation is the most often used method since it is simple, easy, ecologically beneficial, has excellent purity, no agglomeration, and low calcination temperature^4,6^. The characteristics of these adaptable materials are influenced by several factors, including the technique, preparation circumstances, parameters, replacing cations, and cation distribution, as well as the controlled analytical synthesis environment and characterisation^3^. Hard ferrimagnetic materials are cobalt ferrites (CoFe_2_O_4_). They possess an easily assimilated structure and are amenable to synthesis. They possess a significant magnetocrystalline structure, a broader coercivity, a medium magnetic saturation, chemical stability, mechanical strength, and a high electrical resistivity^7^. The ferrite of cobalt CoFe_2_O_4_is a hard ferrite with a slightly inverted spinel structure. The semi-hard cobalt nano-ferrites are a subclass of ferrites. The coercive, stable, robust, crystalline, and resistive characteristics of mid-hard cobalt ferrites^8^. The octahedral position has more Co^2+^ ions, while the tetrahedral site contains more Fe^3+^ ions. The magnetic moment of Fe^3+^ ions is 5µ_B_ and that of Co^2+^ ions is 3 µ_B_^9^. Cu-ferrite is an inverted spinel p-type semiconducting material^5^. Copper (Cu^2+^) cations play a beneficial function in ferrite materials due to their high ductility and low hardness single crystal, as well as their Jahn-Teller effect^10^. Magnesium ferrite is one of the most important ferrites with an inverse cubic structure, and it is a soft ferrite n-type semiconducting material with several uses in magnetics, heterogeneous catalysis adsorption, and sensors. Due to the diamagnetic characteristic of Mg; Mg- ferrite may have relatively low magnetic anisotropy^5^. Numerous studies on Mg doping in various ferrite matrices have revealed that Mg doping has a significant influence on electrical, magnetic, and morphological properties. Because of its great thermal and chemical durability, magnesium oxide may be employed in a variety of technical applications in severe settings^11^. Due to the variability of individual Mg- and Cu- ferrites, magnesium-copper (Mg-Cu) ferrite appears to be extremely fascinating among distinct ferrites. Spinel structures may accommodate a variety of cations with varying ionic sizes without generating significant structural distortion. Many cations may be easily replaced into the spinel structure’s tetrahedral and octahedral positions. The magnetic characteristics of spinel ferrites are affected by changes in cation distribution^12^.

Parajuli et al^7^. synthesized Co_0.5_M_0.5−x_Cu_x_Fe_2_O_4_ nano ferrites using the sol-gel method involving auto-combustion. The results showed spinel structures with decreasing lattice constants with concentration. The crystallite size increased with attention, while the lattice parameter decreased with increasing Cu^2+^ content. FESEM showed grain sizes first increase and reduce Co-Mg-Cu composition, then decrease and increase in Co-Zn-Cu with increasing Cu^2+^ cations. FTIR showed an increasing trend of transmittance with concentration. The canting impact on the semi-disordered system, like Co-Zn ferrite, decreased magnetization. The activation energy decreased with conducting Copper concentration, with Co-Zn-Cu showing a semiconducting behavior.

Muhammad Hadi et al^13^. synthesized nanoparticles and doped Cu-doped Co-Zn ferrites using auto-combustion sol-gel synthesis. X-ray diffraction confirmed the single-phase structure, while transmission electron microscopy and area electron diffraction patterns confirmed the polycrystalline nature. Energy-dispersive X-ray spectroscopy revealed the elemental composition and formation of a ferrite composite. Dielectric spectroscopy showed Maxwell-Wagner interfacial polarization, which decreased with frequency. The samples can be used for multilayer inductor chips, with increased ac conductivity and frequency. Sabih et al. discovered that Ce and Zn substituted Co-Cu ferrites exhibit soft magnetic behavior, making them suitable for applications such as water treatment, recording, and memory^14^.

In this work, the Co-precipitation method was used to synthesize the nano-ferrite Co_0.5_Mg_x_Cu_(0.5−x)_Fe_2_O_4_ system where (x = 0.0, 0.1, 0.2, 0.3, and 0.4) and investigate the effects of Mg substitution on the structural, mechanical, and magnetic properties by using XRD, EDX, FT-IR, TEM, and VSM techniques. Magnesium replacement has impacted the structural, electrical, and magnetic properties of many ferrite systems. According to the literature survey, no report has been made about the effect of Mg substitution for Co-Cu nano-ferrite to investigate the structure, the characteristics, and the magnetic properties. The earlier study looked at what happens when Co^2+^ and Cu^2+^ ions are swapped out in ferrite nanoparticles (NPs) with the formula Mn_0.4_Co_(0.6−x)_Cu_x_Fe_2_O_4_. It looked at how these changes affect the NPs’ structure, shape, and magnetic. Doping with Cu^2+^ ions in MnFe_2_O_4_ NPs resulted in a slight increase in magnetization, whereas doping with Co^2+^ ions resulted in a significant increase in magnetization. Doping MnFe_2_O_4_ with Co^2+^ and Cu^2+^ions increased the surface area, confirming the enhancement of the magnetic properties of ternary-doped ferrite NPs compared to their pristine counterparts^15^.

## Experimental

### Materials and synthesis of Co0.5MgxCu0.5-xFe2O4 nanoparticles

The required samples with the chemical formula Co_0.5_Mg_x_Cu_0.5−x_Fe_2_O_4_ (x = 0.0, 0.1, 0.2, 0.3, and 0.4) were synthesized in this investigation using the wet-chemical co-precipitation technique. The following materials and components are needed for the experiment (as Table 1): Ferric Chloride [FeCl_3_], Cobalt (ll) Nitrate Hexahydrate [Co(NO_3_)_2_.6H_2_O], Magnesium Nitrate Hexahydrate [Mg(NO_3_)_2_.6H_2_O], Copper(ll) Nitrate [Cu(NO_3_)_2_.3H_2_O] and Sodium hydroxide [NaOH] from Laboratory Reagens & Fine Chemicals. High-quality metal nitrates were dissolved in 0.1 M aqueous solutions in a stoichiometric amount and mixed with the starting components using a magnetic stirrer at a lower temperature. To achieve a PH of 10, around 3 M NaOH solution was added drop by drop to the salt solution in an ice bath. Then, the solutions were heated at 80˚C for 2 h until precipitates formed. Deionized water was used to rinse and centrifuge the precipitate. After washing the product with deionized water to remove salts, it was heated at 75˚C for 24 h in a normal atmosphere and crushed to a fine powder in an agate mortar.

**Table 1:** The using weight of each sample.

X	Ferric Chloride [FeCl_3_]	Cobalt (ll) Nitrate Hexahydrate [Co(NO_3_)_2_.6H_2_O]	Copper(ll) Nitrate [Cu(NO_3_)_2_.3H_2_O]	Magnesium Nitrate Hexahydrate [Mg(NO_3_)_2_.6H_2_O]
0	8.1105	3.637875	3.02	0
0.1	8.1105	3.637875	2.416	0.641025
0.2	8.1105	3.637875	1.812	1.28205
0.3	8.1105	3.637875	1.208	1.923075
0.4	8.1105	3.637875	0.604	2.5641

### Characterization and properties of Co0.5MgxCu0.5-xFe2O4 nanoparticles

The sample’s crystal structure was determined by X-ray diffraction (using a Philips model (PW- 1729) diffractometer Cu-Kα radiation wavelength 0.1540598 nm with scattering angle 2θ ranging from 10˚ to 80˚. Fourier transform infrared spectroscopy (FTIR, PERKIN-ELMER-1430) was used to assess the samples for absorption band and functional group information from 200 to 2000 cm^−1^, the very small amount of sample was mixed with KBr to prepare a transparent tablet at room temperature. The morphology of nanomaterial was investigated using a high-resolution transmission electron microscope (HRTEM assessment carried in a 200 kV-operated JEOL- JEM-2100), the very small amount of sample was substituted in Dimethylformamide (DMF) and then used in grades to take photos. Energy-dispersive X-ray (EDX) spectroscopy (JOEL, Model: JSM-5200 LV) was used to determine the elemental content of samples, the very small amount of sample was used as a powder to take ESM photos, and then use computer program of the SEM to analyze the photos. A vibrating sample magnetometer (lab-built) was used to determine the magnetic characteristics of the samples, the details of the measuring technique had been reported in the previous work^16^.

## Results and discussion

### Structural analysis

**Figure 1:**
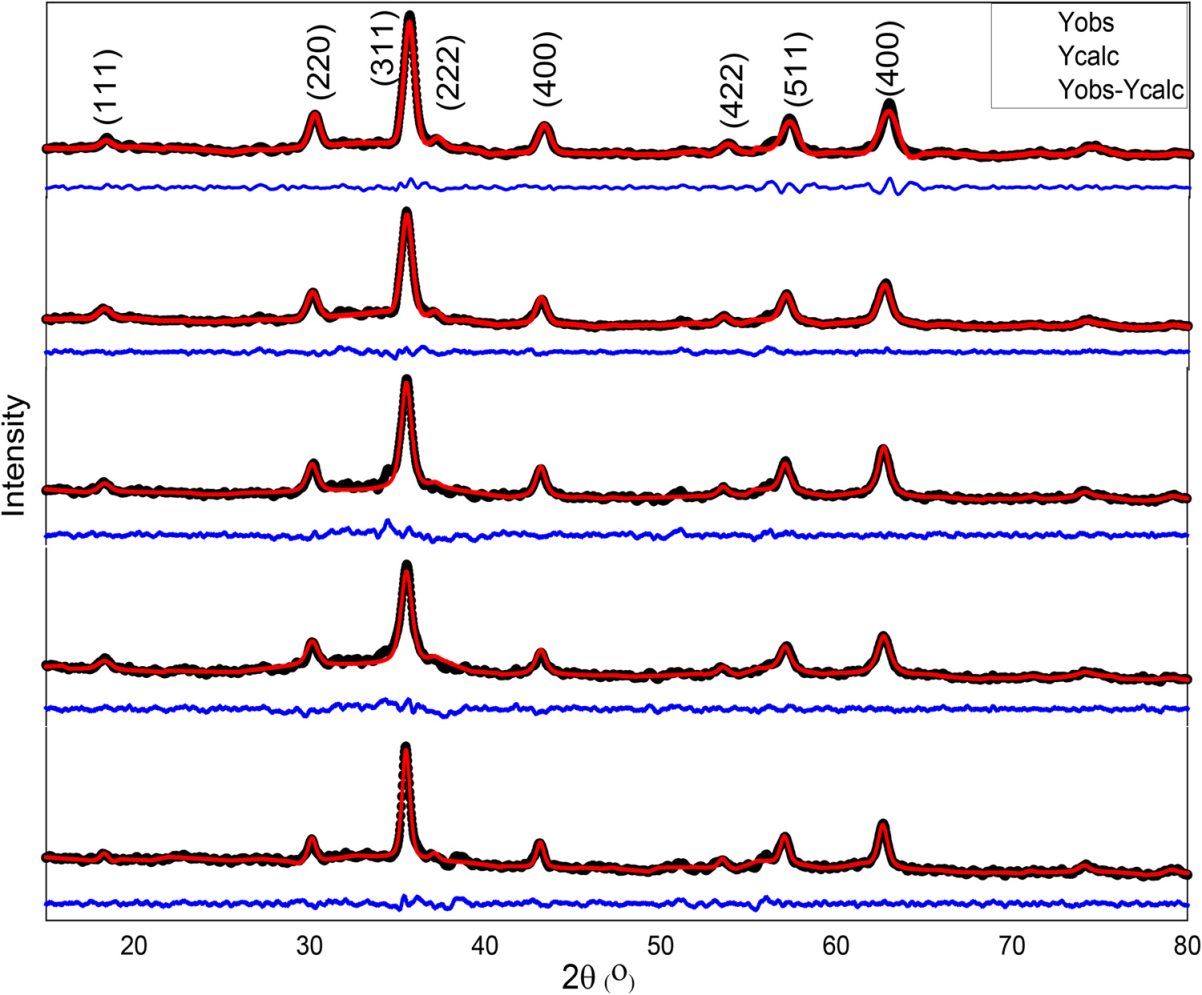
Rietveld fitting of X-ray diffraction of Co_0.5_Mg_x_Cu_0.5−x_Fe_2_O_4_ (x = 0, 0.1, 0.2, 0.3 and o.4) nano-ferrite sample.

Figure 1 depicts the XRD patterns of Co_0.5_Mg_x_Cu_0.5-x_Fe_2_O_4_ nano-ferrite samples with x = 0.0, 0.1, 0.2, 0.3, and 0.4, and the Rietveld fitting of XRD. The observed peaks (111), (220), (311), (400), (511), and (440) verified the creation of a single-phase cubic spinel structure with no additional phases when compared to the JCPDS card numbers 22-1086, 88-1937, and 77 − 0010 for cobalt, magnesium, and copper ferrite, respectively. The amorphous nature of the Co_0.5_Mg_x_Cu_0.5-x_Fe_2_O_4_ samples under study is demonstrated by the broadening of XRD peaks. The most notable peak (311) shows a shifting towards lower 2θ values as Mg^2+^ content in Co_0.5_Mg_x_Cu_0.5-x_Fe_2_O_4_ increases which indicates the corresponding increase in the lattice parameter (*a*) as shown in the inset of Fig. 1.

The experimental lattice parameter (*a*_exp_) was estimated using the following Eq. (1):

1 $$\:{\mathbf{a}}_{\mathbf{e}\mathbf{x}\mathbf{p}}=\mathbf{d}\sqrt{{\mathbf{h}}^{2}+{\mathbf{k}}^{2}+{\mathbf{l}}^{2}}\:\:\:$$

where (*d*) is the Interplanar spacing and *(hkl)* are Miller indices. Peak sharpness links the nano range of crystallite sizes as illustrated by Debye Scherer’s equation. For the (311) peak, the Debye Eq. (2) is utilized to determine the crystallite sizes (*R*) of all samples.

2 $$\:\mathbf{R}=\frac{\mathbf{k}\:\varvec{\uplambda\:}}{\varvec{\upbeta\:}\mathbf{c}\mathbf{o}\mathbf{s}\varvec{\uptheta\:}}$$

where (k) is the Scherer factor, (λ) is the wavelength of Cu k α radiation, (β) is Full width at half maximum of the peak (311), and (θ) is the angle of diffraction.

**Figure 2:**
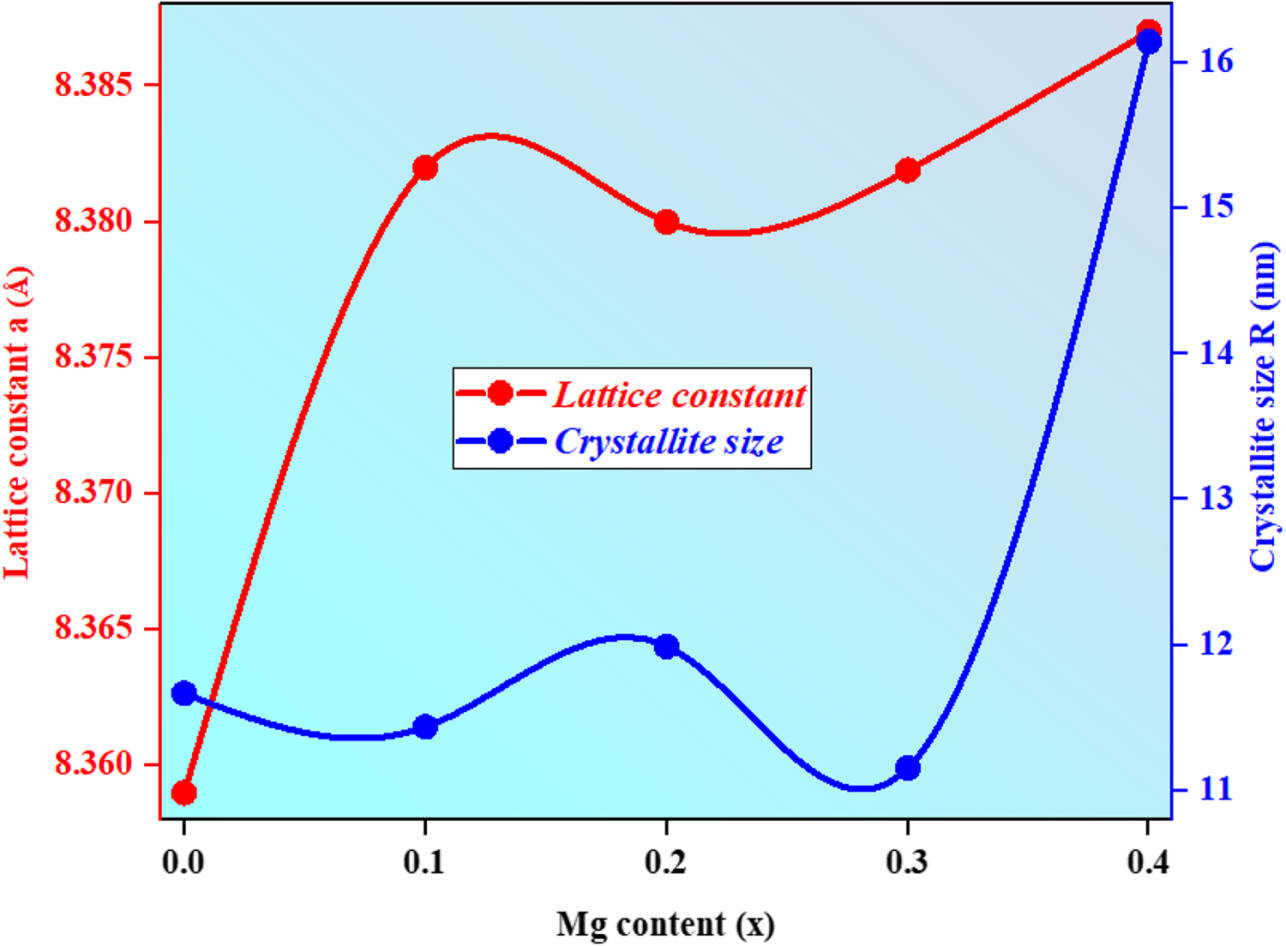
Variation of the lattice constant (**a**) and the crystallite size (R) with Mg content.

Figure 2 depicts the trend of lattice constant and crystallite size with Mg^2+^ content. It indicates that when Mg^2+^ doping increased according to Vegard’s law, the sample lattice constants were in the range between (8.359 to 8.387Å). A little rise in lattice constants may result from the partial inverse spinel ferrite substitution process between Mg^2+^ (0.72) and Cu^2+^ (0.73). Additionally, the Jahn-Teller distortion responsible for the observed shift in the lattice parameter occurs according to the Cu^+2^ existence. The Jahn-Teller distortion reduces and the cubic symmetry increases with increasing concentration of Mg^2+^ ions. According to X-ray confirmation, the Jahn-Teller distortion observed in the sample could represent a compression distortion at the octahedral B site. As Mg^2+^increases, the octahedral site reverts to its symmetry in the cubic form^17^. The crystallite sizes were in the range between (11.1570 and 16.1457 nm) which demonstrates that the ferrite generated is nanocrystalline, it decreases with increasing Mg content due to the substitution process between Mg^2+^ (0.72) and Cu^2+^(0.73) and the increase in stain lattice confirm this behavior except at x = 0.4, it increases may be due to the Jahn-Teller distortion^17^.

Several direct and indirect causes affect variations in the lattice constant, perhaps causing an increase rather than a decrease, according to Rietveld’s study. While there are less than two cations occupying (B) sites, there are more cations occupying (A) sites. The number of cations occupying (A) sites falls as the Mg content rises, whereas the number filling B sites grows, raising the lattice constant. Co cations transfer to (B) sites at x = 0.3, while most Co^2+^ cations normally inhabit (A) sites. The contribution of magnesium varied at the (A) and (B) sites, even though it replaced Cu cations in identical ratios. For example, when comparing the (A) sites’ 0.037 Cu outlets and 0.026 Mg inlets to the conventional x = 0.1, the B sites have 0.06 Cu outlets and 0.075 inlets. The Mg/Cu confinement from (B) sites to (A) sites causes the lattice constants to rise.

The lattice strain **(ε)** which is calculated using the following Eq. (3), offers further information about the structural characteristics of Co_0.5_Mg_x_Cu_0.5-x_Fe_2_O_4_ as displayed in Table 2. According to the calculations, the strain had a greater impact on the sample morphology than the flaws and imperfections (voids, interstitials, and vacancies) inside the crystal lattice.

**Table 2:** XRD parameters: concentration (X), lattice constant *a* (Å), Crystallite size R (nm), particle size from TEM < D > _TEM_ (nm), dislocation line density δ (10^−3^nm^−2^), the lattice strain ε, X-ray density D _x_ (gm/cm^3^), bulk density D _b_ (gm/cm^3^), and Porosity p (%)Co_0.5_ Mg_x_Cu_0.5−x_Fe_2_O_4_ (x = 0, 0.1, 0.2, 0.3 and 0.4) nano ferrite particles.

X	a (Å)	*R* (nm)	< D > _TEM_ (nm)	δ (10^−3^nm^−2^)	Ε	D _x_ (gm/cm^3^)	D _b_(gm/cm^3^)	*p* (%)
0.0	8.359	11.6690	10.79	7.3439	9.72E-03	5.481	2.7263	50.258
0.1	8.382	11.4398	12.43	7.6412	9.94E-03	5.735	2.8878	49.644
0.2	8.38	11.9925	12.66	6.953	9.48E-03	5.238	2.7584	47.337
0.3	8.3819	11.1570	11.59	8.0333	1.02E-02	5.442	2.8291	48.012
0.4	8.387	16.1457	16.8	3.8360	7.05E-03	5.584	2.7550	50.661

3 $$\epsilon=\frac{\beta\cos\theta}{4\sin\theta}$$

Furthermore, the lattice parameter is utilized to compute the unit cell volume **(V)** of Co_0.5_ Mg_x_Cu_0.5-x_Fe_2_O_4_ samples using the volume-lattice parameter relationship **V = a**^**3**^, It is closely tied to the lattice parameter, so it follows the same trend. A dislocation is a crystallographic flaw that has a significant impact on material characteristics. Dislocations are one-dimensional crystalline flaws that disturb the regular atomic array of a flawless crystal^6^. The dislocation line density may be calculated using the following Eq. (4) based on the average crystallite size.

4 $$\:\mathsf{\delta\:}=\frac{1}{{\varvec{R}}^{2}}$$

Table 2shows that the obtained dislocation densities are lower, implying that the Nano-ferrite particles are more crystalline^18^. Table 2 provides a summary of structural parameters.

Also, X-ray density is estimated using the following Eq. (5), and the results are shown in Table 2.

5 $$\:{\mathbf{D}}_{\mathbf{x}}=\frac{\mathbf{Z}\mathbf{M}}{\mathbf{N}\mathbf{A}{\mathbf{a}}^{3}}$$

Where (Z) is the number of molecules in a unit cell = 8, (M) is the Molecular weight of the samples, (N_A_) Avogadro’s number, (a^3^) is the volume of the unit cell. The bulk density ($\:{D}_{b}$) is calculated from the following Eq. (6)

6 $$\:{\varvec{D}}_{\varvec{b}}=\frac{\varvec{m}}{\varvec{\pi\:}{\varvec{r}}^{2\:}\varvec{t}}$$

**Figure 3:**
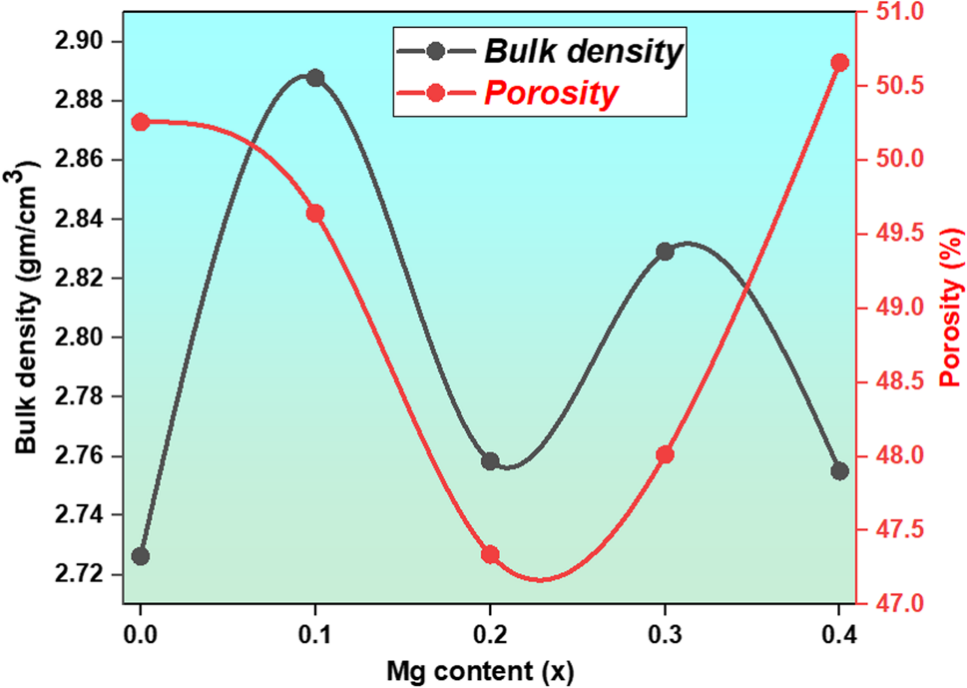
Variation of the bulk density and porosity with Mg content.

Where (m) is the mass of the sample, (r) is the sample radius, (t) is the thickness of the sample. With the aid of the relation $\:\varvec{p}=(1-\frac{{\varvec{D}}_{\varvec{b}}}{\:{\varvec{D}}_{\varvec{x}}})\:\varvec{\%}$ porosity provides empty spaces or voids in the substance. (D_b_) is the bulk density and (D_x_) is the x-ray density. The lattice parameter and molecular weight act as dual variables influencing sample density, with D_X_ being inversely proportional to the lattice parameter and directly proportional to the molecular weight. The irregular behavior of the lattice parameter and the irregular molecular weight (from the substitution of lighter Mg (24.305 g/mole) ions at the expense of Cu (63.546 g/mole) ions) influence the D_x_ behavior, as seen in Table 2. Meanwhile, D_b_ displays aberrant behavior with lower values than D_x_due to pore formation during the preparation method^18^. Porosity is related to D_x_ and D_b_ values. Porosity declines as Mg^2+^ concentrations rise, with nano ferrite having the lowest at x = 0.4; as shown in Table 2. Moreover, the porosity behavior may be explained in terms of the inverse relationship between crystallite size and porosity, since as crystallite size declines, the chance of pore formation rises^18^, as shown in Table 2. The porosity trend is seen in Fig. 3, Always find the porosity of bulk ferrites is in the range of 20–30%, while the porosity of nano-ferrites is in the range of 50–80%, depending on the grain or crystallite size. Many factors, such as grains, grain boundaries, agglomeration, and imperfections, contribute to porosity. These reasons diminish as the annealing temperature increases and may disappear at very high sending temperatures for single crystals. In this research, the prepared temperature is 80 °C with no annealing, calcination, or sintering. The prepared materials are nanomaterials in the range of 11–16 nm, according to TEM measurements.

The physical and chemical characteristics of spinels can change depending on where the cations are in the crystal lattice. More specifically, it is determined by cation occupancy at octahedral/tetrahedral lattice locations^18^. Metal cation distribution laws are often connected to characteristics such as ion radius, electron layer structure, ion valence bond balance, ion ordering, and so on. The suggested cation distribution for the tetrahedral (A) and octahedral (B) sites according to Rietveld analysis is shown in Table 3. The metal ions of Fe, Mg, and Cu are preferred to occupy the octahedral (B) site, while the metallic ion of Co is preferred to occupy the tetrahedral (A). The structural refinement obtained parameter by the Rietveld method are listed in Table 4.

**Table 3:** The cation distribution of Co_0.5_ Mg_x_Cu_0.5−x_Fe_2_O_4_ (x = 0, 0.1, 0.2, 0.3, and 0.4) nano ferrite samples according to Rietveld analysis.

X	Chemical composition	Cation distribution
0.0	Co_0.5_ Cu_0.5_Fe_2_O_4_	(Fe_0.59_ Co_0.34_Cu_0.14_)_A_ [Co_0.15_Cu_0.35_Fe_1.4_]_B_ O_4_
0.1	Co_0.5_ Mg _0.1_ Cu_0.4_Fe_2_O_4_	(Fe_0.59_ Co_0.35_Mg_0.02_Cu_0.1_)_A_ [Co_0.14_Mg_0.07_Cu_0.29_Fe_1.4_]_B_ O_4_
o.2	Co_0.5_ Mg _0.2_ Cu_0.3_ Fe_2_O_4_	(Fe_0.59_ Co_0.35_Mg_0.05_Cu_0.08_)_A_ [Co_0.14_Mg_0.14_Cu_0.21_Fe_1.4_]_B_ O_4_
0.3	Co_0.5_ Mg _0.3_ Cu_0.2_Fe_2_O_4_	(Fe_0.59_ Co_0.24_Mg_0.07_Cu_0.05_)_A_ [Co_0.25_Mg_0.22_Cu_0.14_Fe_1.4_]_B_ O_4_
0.4	Co_0.5_ Mg _0.4_ Cu_0.1_Fe_2_O_4_	(Fe_0.61_ Co_0.31_Mg_0.1_Cu_0.02_)_A_ [Co_0.18_Mg_0.29_Cu_0.07_Fe_1.3_]_B_ O_4_

**Table 4:** Structural parameters for Co_0.5_ Mg_x_Cu_0.5−x_Fe_2_O_4_ ferrite obtained the structural refinement by the Rietveld method.

Sample	*r*_A_(Å)	*r*_B_(Å)	X^2^	Bragg *R*-factor	RF-factor	Rwp	Rexp
Co-Cu	0.657	0.5709	0.186	3.828	2.224	17.1	39.63
Co-Cu-Mg1	0.651	0.5734	0.2	2.72	2.198	17.5	39.13
Co-Cu-Mg2	0.654	0.5714	0.263	8.748	5.495	25.3	49.26
Co-Cu-Mg3	0.572	0.6119	0.236	11.7	7.287	27.5	56.51
Co-Cu-Mg4	0.623	0.5856	0.169	7.204	6.895	26.1	63.47

### Fourier transform infrared spectroscopy (FTIR)

**Figure 4:**
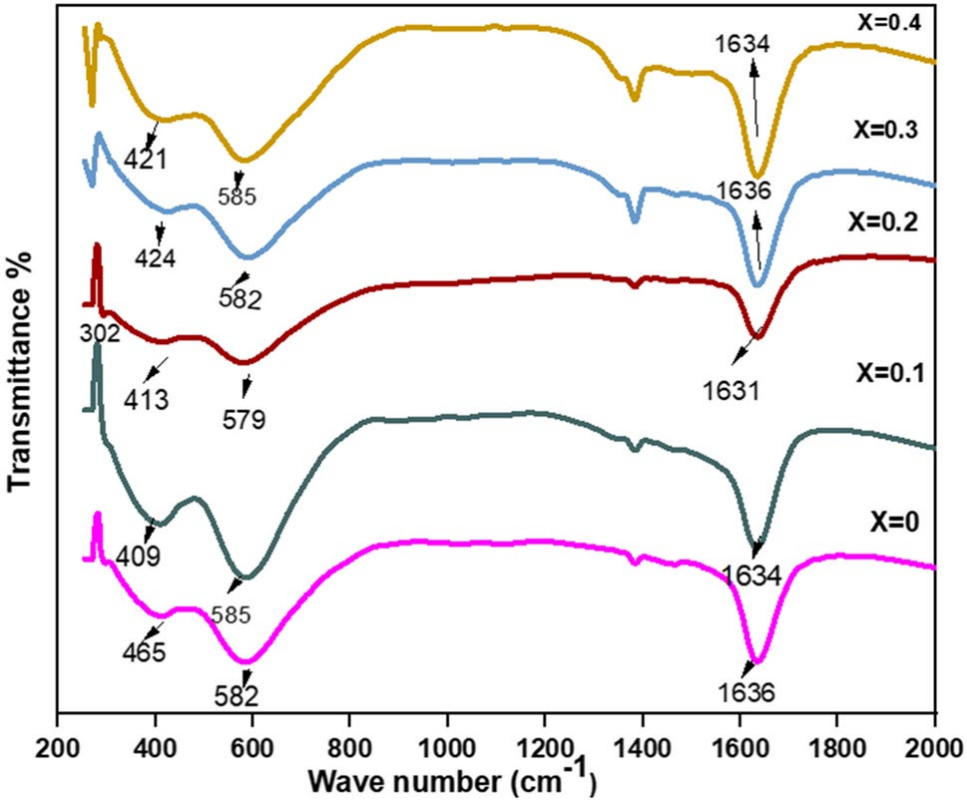
FTIR spectra of Co_0.5_ Mg_x_Cu_0.5−x_Fe_2_O_4_ (x = 0, 0.1, 0.2, 0.3 and 0.4) nano ferrite samples.

The FTIR spectra of Co_0.5_ Mg_x_Cu_0.5−x_Fe_2_O_4_ samples from 200 to 2000 cm^−1^ are shown in Fig. 4 and listed in Table 5, they depict two prominent vibrational mode frequencies, ʋ_1,_ and ʋ_2_, which are in the ranges of (580–588 cm^−1^) and (409–465 cm^−1^) respectively, the vibration of tetrahedral and octahedral components in spinel ferrites is responsible for the difference in band position^19^. The detected vibrational mode frequencies are all smaller than 1000 cm^−1^, showing that the spinel structure is generated by oxygen ion lattice vibrations against metal ions. All synthesized samples are characterized as spinel structures as a consequence of Figs. 4^6,20^. The band ʋ_1_is related to stretching vibrations of tetrahedral site metal ion and oxygen bonding, while ʋ_2_can be assigned to the divalent metal ion-oxygen complexes among the octahedral sites^21^. The possibility of the existence of divalent metallic bonds Mg^2+^-O^2−^, Cu^2+^-O^2−^ and/or Fe^2+^-O^2−^ in octahedral sites explains the appearance of the absorption band ʋ^3^ (302.31 cm^−1^) only at x = 0.2^22^. While the absorption band ʋ_4_ (292.96 cm^−1^ and 269.69 cm^−1^) only at x = 0.3 and 0.4 depends on the mass of the divalent tetrahedral cation and is ascribed to some form of lattice vibration including a displacement of the tetrahedral cation^22,23^. The spectra indicated a band of about 1400 cm^−1^ and a large band of about 1600 cm^−1^these were ascribed to free or absorbed water H-O-H bending vibrations^4^. This residual water in the Nano ferrites might be due to the preparation method^21^. From Fig. 4 addition to Mg^2+^ substitution, the values of bands ʋ_1_ and ʋ_2_ and their broadening are varied. This behavior is connected to cation distribution between interstitial (tetrahedral and octahedral) sites, which resulted in cation-oxygen ion bond length variations. These variations in bond length are the primary causes of the shifts in frequencies ʋ_1_ and ʋ_2_^10^.

**Table 5:** FTIR vibrational frequencies and force constants of Co_0.5_ Mg_x_Cu_0.5-x_Fe_2_O_4_ nano-ferrite.

X	ʋ_1_ (cm^−1^)	ʋ_2_ (cm^−1^)	F_A_ *10^5^ (dyne/cm)	F_B_ *10^5^ (dyne/cm)	F_avg_ *10^5^ (dyne/cm)	θ_D_ (K)
0	584.57	465.71	2.504395	1.589501	2.046948	755.15132
0.1	585.79	409.97	2.514859	1.231782	1.873321	715.95144
0.2	580.42	413.97	2.468963	1.255936	1.862449	714.96641
0.3	588.69	424.88	2.539821	1.323008	1.931414	728.75683
0.4	582.9	421.79	2.490106	1.303834	1.896970	722.37211

#### Force constants

The force constant parameters for the Fe^3+^- O^2−^ bond at tetrahedral and octahedral sites were computed by inserting the values of ʋ_1_ and ʋ_2_ vibrational frequencies into the following Eq. (7):

7 $$F=4\pi^2C^2\upsilon^2\mu$$

**Figure 5:**
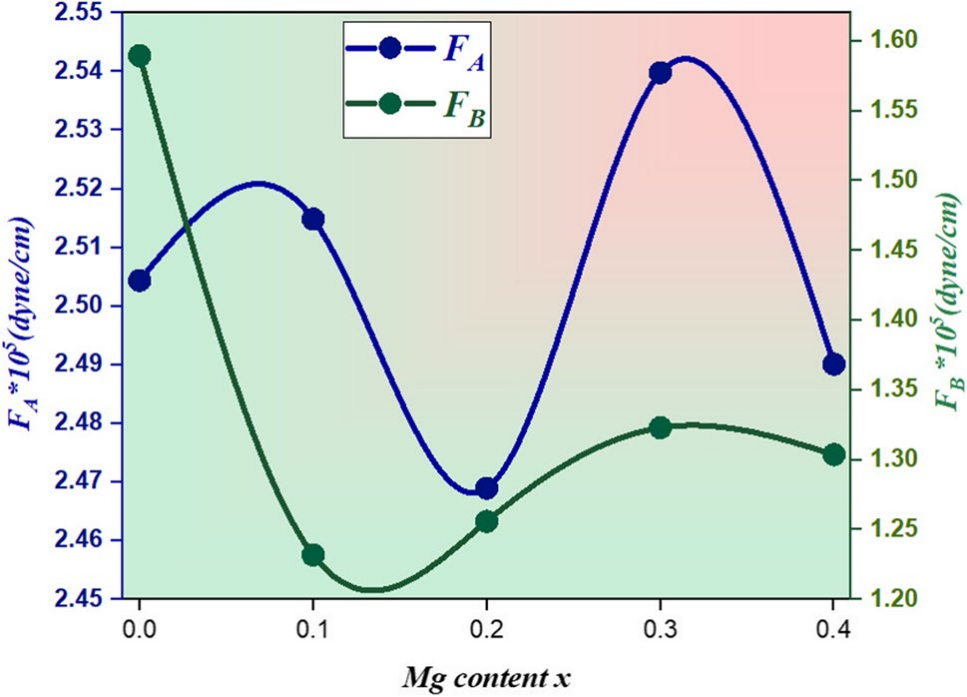
The relationship between the force constant FA, FB of Co_0.5_ Mg_x_Cu_0.5-x_Fe_2_O_4_, and Mg content x.

Where (ʋ) is the Frequency of vibration, (C) is the Velocity of light (= 3*10^8^ m/s), and (µ) is reduced mass which is calculated according to the cation distribution. Figure 5 illustrates the relationship between the force constant F_A_, F_B_ of Co_0.5_Mg_x_Cu_0.5−x_Fe_2_O_4,_ and Mg content x which is listed in Table 5. The difference in frequency between the characteristic vibrations ʋ_1_ and ʋ_2_ can be attributed to metal - O^2−^ ions in the tetrahedral and octahedral sites, respectively. The overall trend shows that the force constant of the tetrahedral site (F_A_) is found to be bigger than that of the octahedral site (F_B_); this might be due to stronger covalent bonds in the tetrahedral site than in the octahedral site, and stretching in the tetrahedral site results in a higher force constant for the tetrahedral site than for the octahedral site^21^.

#### Debye temperature

The Debye temperature θ_D_ can be calculated using the following Eq. (8):

8 $${\boldsymbol\theta}_{\mathbf D}\boldsymbol=\frac{\left(\boldsymbol{\hbar}\mathbf C{\boldsymbol\upsilon}_{\boldsymbol a\boldsymbol v\boldsymbol g}\right)}{\mathbf K}$$

Where: $\hbar = {h}/{2\pi}$, (h) is the planks constant, (C) is the velocity of light, (ʋ_avg_) is the average wave number, (k) is Boltzmann’s constant, and the value of (ℏC/k) for the spinel ferrite materials is taken as 1.438. Table 5 displays the values of θ_D_ which are found in the range (714.96- 755.15 k), it is found that θ_D_ decreases with increasing Mg^2+^ content until x = 0.2. The reduction in θ_D_ values was accompanied by a reduction in vibrational frequencies and interatomic binding strength. The reduction in θ_D_ values implies that increasing Mg^2+^concentration promotes lattice vibrations, Similar result was reported by A. Bagade, P. Nagwade, A. Nagawade et al^6^.. Another explanation of θ_D_ is the specific heat theory. Specific heat theory may be used to explain the change in Debye temperature θ_D_. The samples’ conduction electrons (i.e., n-type) can absorb some of the heat, resulting in a drop in θ_D_ for $\:\text{x}\le\:0.2$ The rise in θ_D_ for $\:\text{x}>0.2$ might be attributable to a decrease in conduction electrons and an increase in conduction holes (i.e., p-type). This demonstrates that when Mg^2+^ concentration increases, sample conduction shifts from p-type to n-type. This θ_D_ variant demonstrates that conduction electrons may contribute to specific heat and therefore to θ_D_^21^.

### Elastic properties

**Figure 6:**
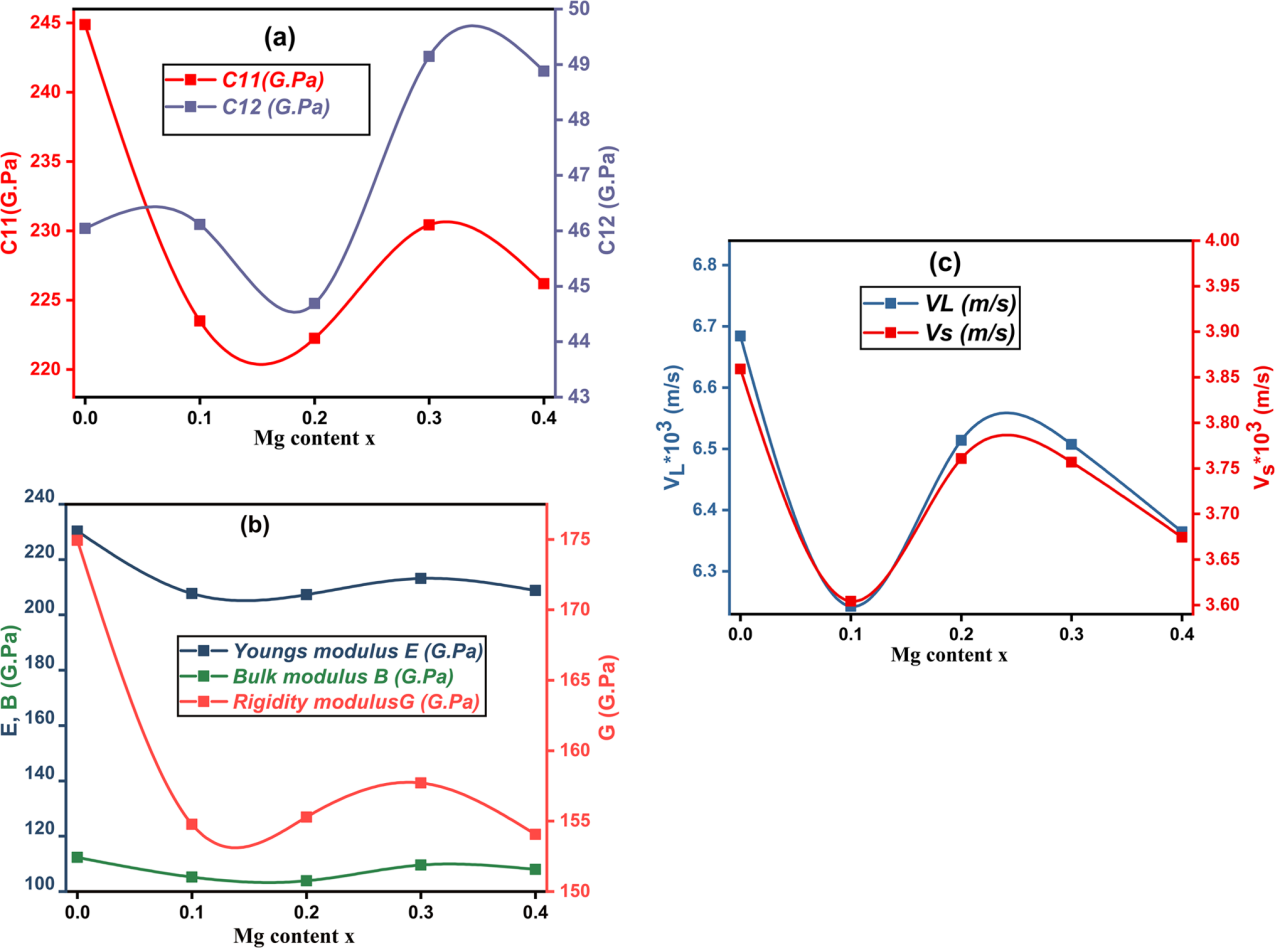
Variation of (**a**) the stiffness constants C_11_ and C_12_, (**b**) Variation of Young’s modulus (**E**), bulk modulus (**B**) and rigidity modulus (**G**) and (**c**) Longitudinal (V_L_) and transverse (V_S_) wave velocities with Mg^2+^ content x in Co_0.5_ Mg_x_Cu_0.5-x_Fe_2_O_4_ (x = 0.0, 0.1, 0.2, 0.3and 0.4) system.

Elastic parameters of ferrite materials may be determined by understanding the various moduli; Young’s (E), bulk (B), longitudinal (L), rigidity (G), and Poisson’s ratio (σ). The data from FTIR spectroscopy may be utilized to calculate the stiffness constant and elastic parameters. The elastic characteristics of ferrite may be estimated by knowing the average values of F_A_ and F_B_. The stiffness constants C_11_ and C_12_ were calculated using the following relations (9):

9 $$\:{C}_{11}=\raisebox{1ex}{${F}_{avg}$}\!\left/\:\!\raisebox{-1ex}{$a$}\right.\:,\:{C}_{12}=\frac{\sigma\:\:{C}_{11}}{1-\sigma\:}$$

where: $\:\left({F}_{avg}\right)$ is the average force constant ($\:{F}_{avg}=\frac{{F}_{A}+{F}_{B}}{2}$), ($\:a)$ is the lattice parameter, and$\:\:\left(\sigma\:\right)$ is Poisson’s ratio which is a function of pore fraction as ($\:\sigma\:=0.324*[1-(1.043*P\left)\right])$^24^. Figure 6 (a) depicts a variation of C_11_ and C_12_ with x content. It shows that C_11_ grows only at x = 0.3 and thereafter drops, whereas C_12_ increases. The rise in C_11_ and C_12_at x = 0.3 might be attributed to the strengthening of inter-atomic bonding^24^. Because stiffness constants are affected by atomic bonding tightness and force constant, the decrease in C_11_ with increasing x content and C_12_ at x = 0.2 may be due to the weakening of the atomic bonding between Fe^3+^ and Mg^2+^ cations. C_12_’s growing trend might be attributed to a drop in force constant and increasing bond lengths^23^. The elastic moduli were calculated using the relations (10):

Young’s modulus: $$\:\varvec{E}=\frac{({\varvec{C}}_{11}-{\varvec{C}}_{12})({\varvec{C}}_{11}+2{\varvec{C}}_{12})}{({\varvec{C}}_{11}+{\varvec{C}}_{12})}$$

Rigidity modulus:

10 $$\:\varvec{G}=\frac{\varvec{E}}{2(\varvec{\sigma\:}+1)}$$

Bulk modulus: $$\:\varvec{K}=\frac{1}{3}({\varvec{C}}_{11}+2{\varvec{C}}_{12})$$

Figure 6 (b) depicts the variance in elastic moduli [B, G, and E] with x contents. Variations can be attributed to increasing Mg^2+^enhancing interatomic binding. Porosity, grain size, lattice flaws, the existence of secondary phases, and other important aspects influence the elastic characteristics of materials^25^. Longitudinal (V_L_) and transverse (or shear) (V_S_) wave velocities were computed using the following equations (11):

11 $$\:{V}_{L}=\sqrt{\frac{{C}_{11}}{{D}_{x}}}\,\&\:{V}_{S}=\frac{{V}_{L}}{\sqrt{3}}$$

Figure 6(c) depicts the relation between them. The transverse sound velocity is found to be lower than the longitudinal sound velocity. Energy is transmitted from particle to particle during wave propagation via a medium through particle vibration. During transverse wave propagation, the vibration of a particle occurs at right angles to the direction of wave transmission, resulting in a larger energy needed to vibrate the neighboring particle. As a result, the wave’s energy is diminished, and the velocity of the transverse wave is lower than that of the longitudinal wave, about half that of the longitudinal wave velocity^24^.

### Transmission Electron microscope (TEM)

**Figure 7:**
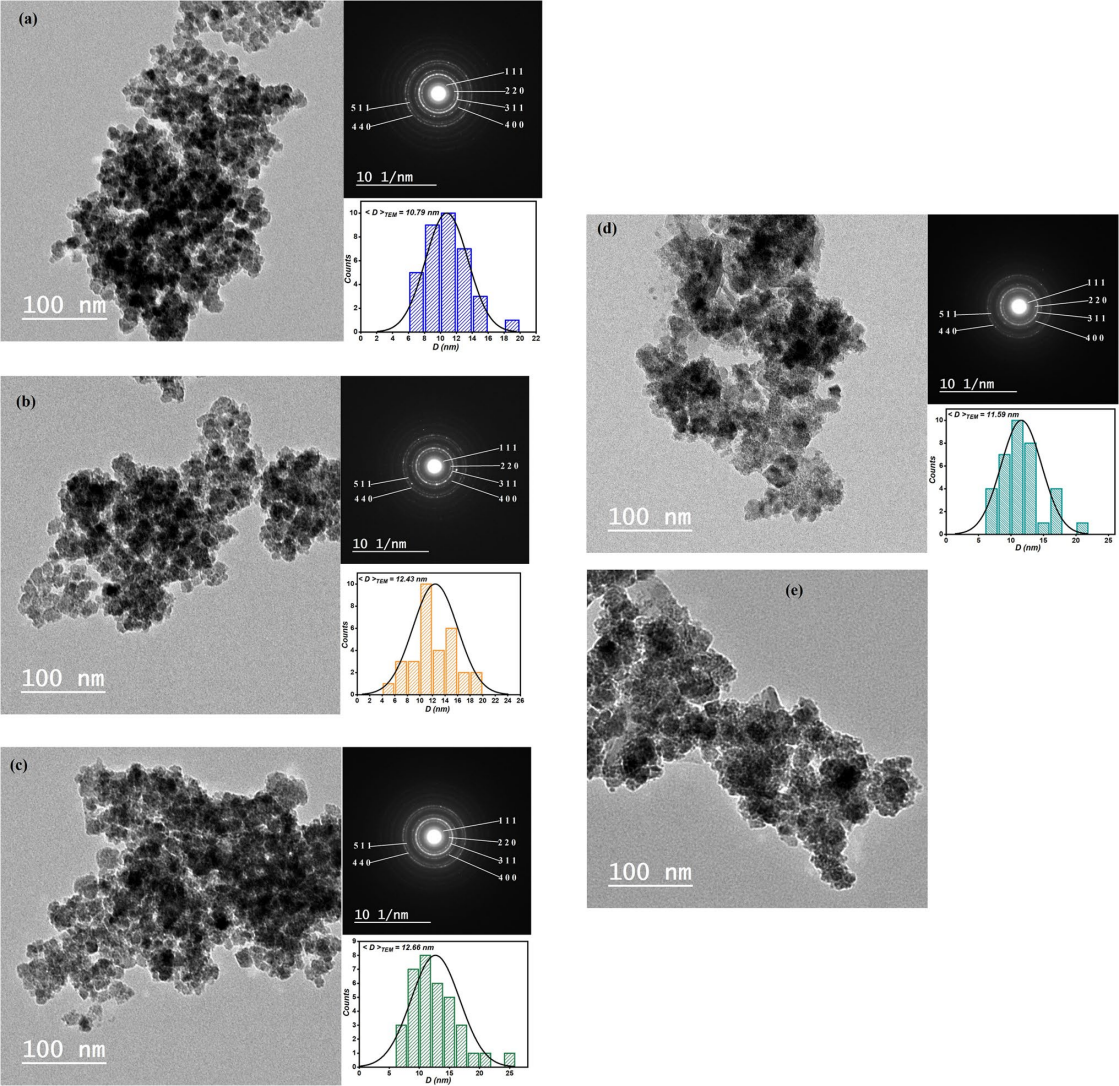
TEM images of Co_0.5_ Mg_x_Cu_0.5−x_Fe_2_O_4_ (x = 0, 0.1, 0.2, 0.3, and 0.4) nano ferrite samples.

Figure 7 shows TEM images for all samples. The images show that the particles are Nano-scale in size and almost spherical, clearly indicating that the preparation procedure produced spherical nanoparticles. The distribution of the particle size in the samples was obtained by determining particle sizes from TEM images. The resulting < D > _TEM_ (as Table 2) values are greater than those obtained from the XRD patterns, which might be attributable to nanoparticle aggregation caused by magnetic interactions between nanoparticles which confirms that the crystallite size for XRD is lower than the grain size from the TEM. Selected area electron diffraction (SAED) patterns of the samples revealed a single crystalline diffraction ring, revealing the spinel structure of the synthesized nanomaterial. The diffraction rings and planes produced from the SAED pattern are connected to (111), (220), (311), (400), (511), and (440) planes, and confirm that the material is polycrystalline. These planes are in great agreement with the values gained from X-ray diffraction patterns.

### Energy dispersive spectroscopy (EDS)

**Figure 8:**
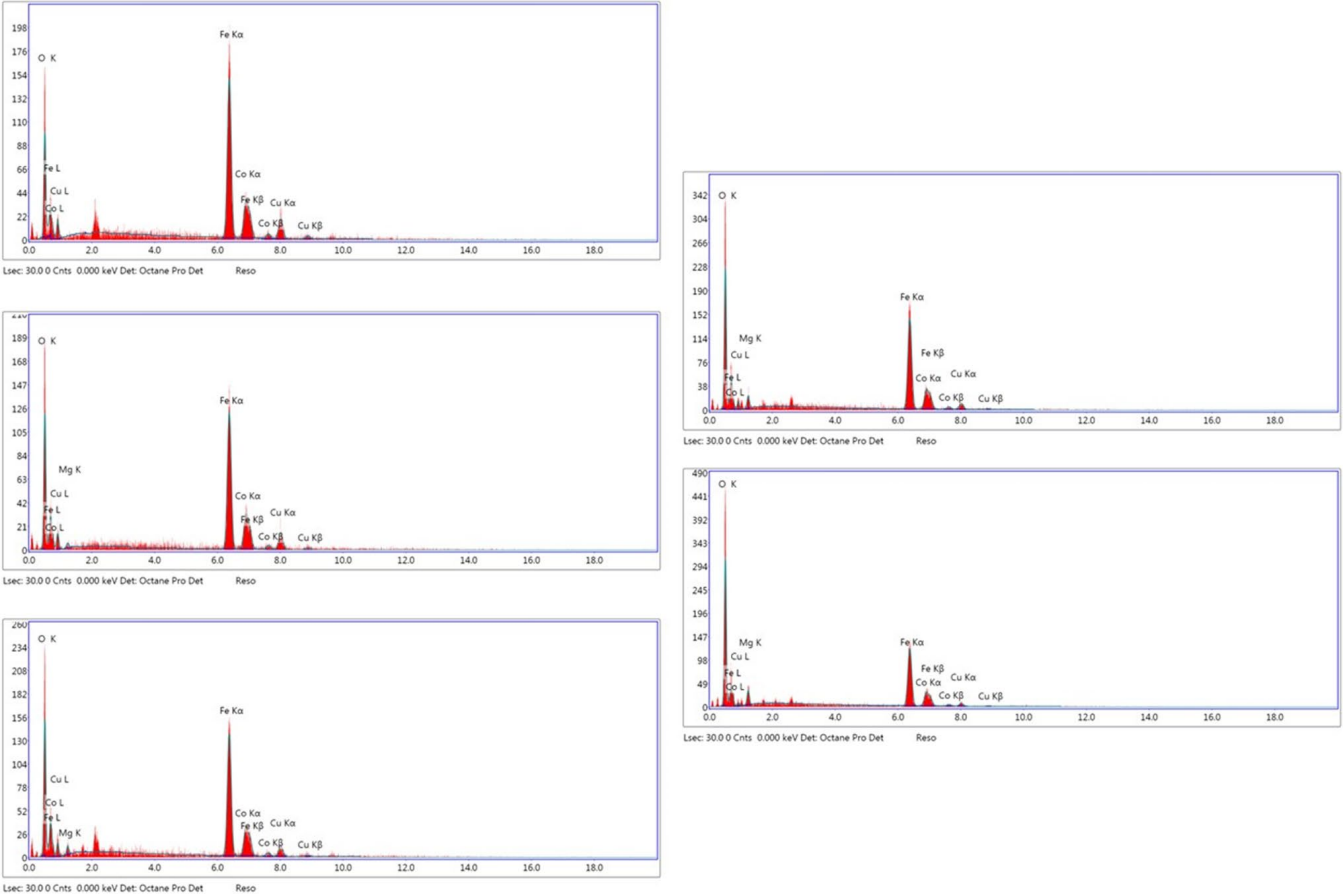
EDX spectrum of Co_0.5_ Mg_x_Cu_0.5−x_Fe_2_O_4_ (x = 0, 0.1, 0.2, 0.3, and 0.4) nano ferrite samples.

The qualitative chemical composition of the samples was studied using energy-dispersive X-ray spectroscopy (EDX), as shown in Fig. 8; Table 6. The EDX data show the presence of all the constituents’ chemical elements (Mg, Co, Cu, Fe, and O) in the final product of Co_0.5_Mg_x_Cu_0.5−x_Fe_2_O_4_ nano-ferrite without any other element Piecemeal increases in Mg content on account of Cu ions, as previously demonstrated by XRD. This confirms that our produced samples are pure Nano-ferrite materials.

**Table 6:** The percentage of the molecular weight of metals in samples.

X	Fe%	Co%	Cu%	Mg%	O%
0.0	52.96	15.02	11.54	0	20.48
0.1	51.28	15.51	11.93	1.09	20.19
0.2	52.59	14.3	8.49	2.06	22.56
0.3	41.93	12.02	5.22	5.47	35.36
0.4	42.14	11.62	3.66	5.21	37.36

### Magnetic measurements

**Figure 9:**
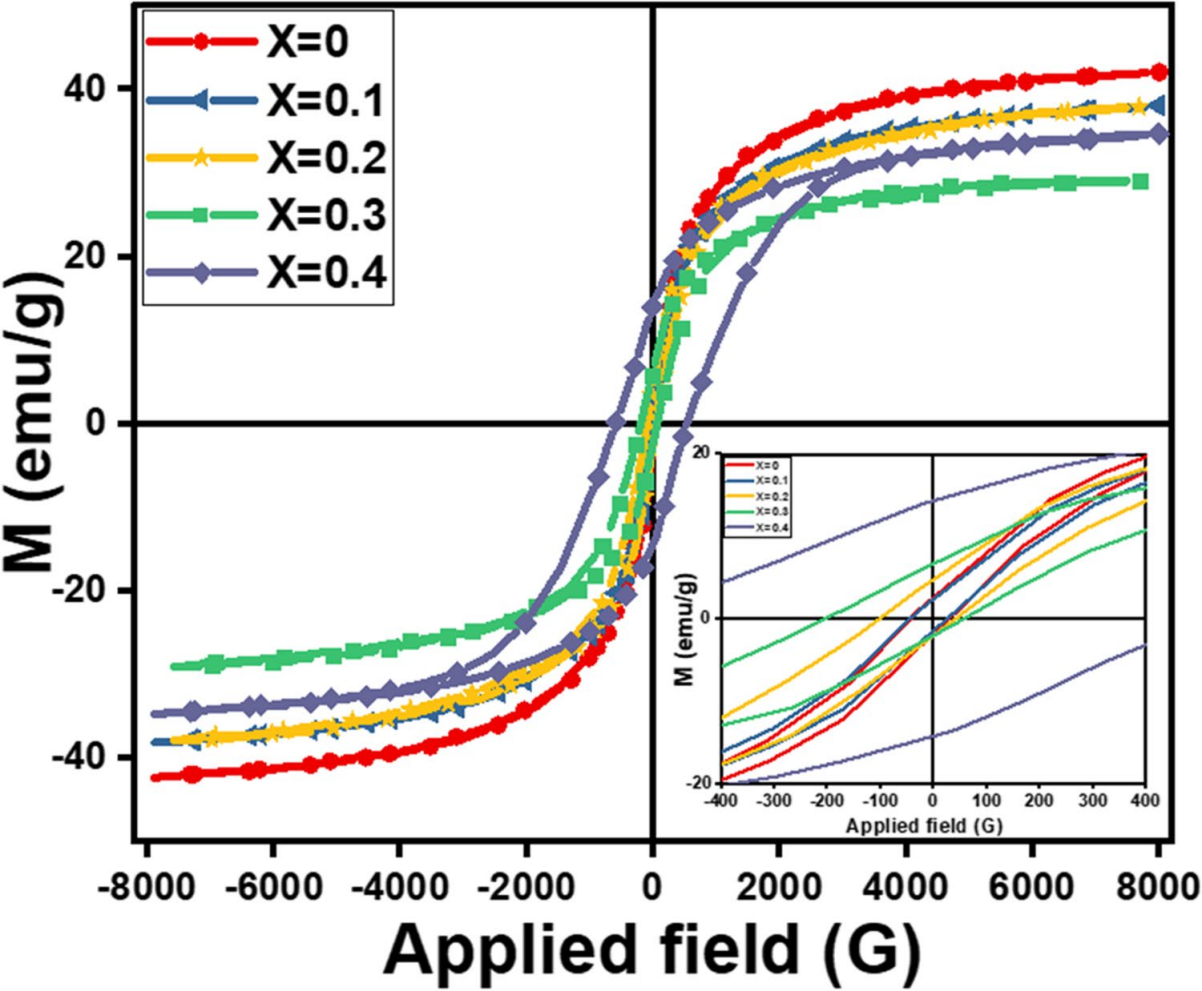
M-H hysteresis loops of Co_0.5_ Mg_x_Cu_0.5-x_Fe_2_O_4_ Ferrite System.

**Figure 10:**
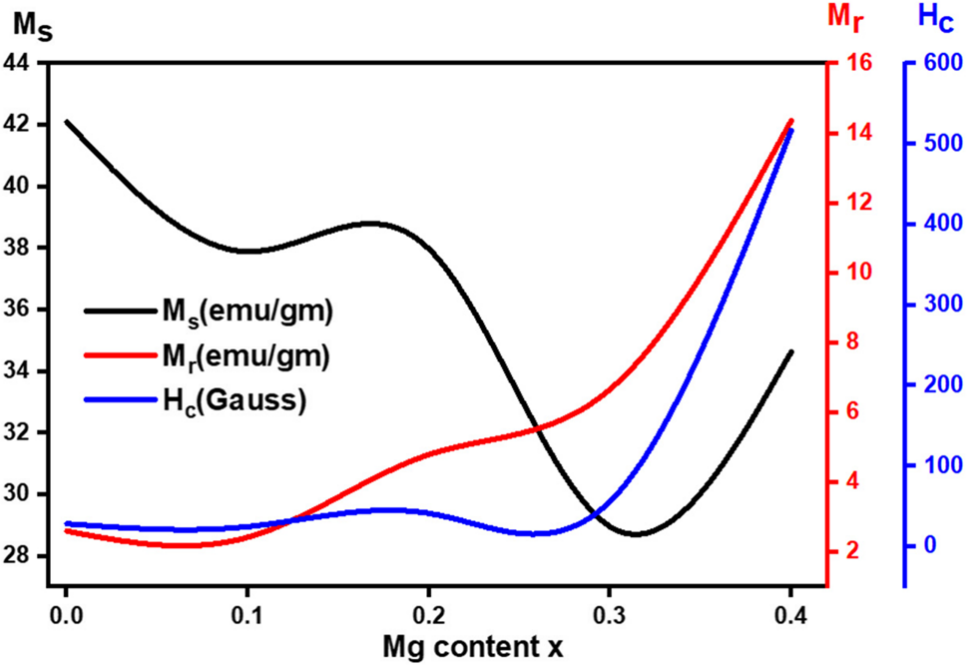
Variation between saturation magnetization (Ms), remanent magnetization (Mr), and coercivity (Hc) with Mg^2+^ content.

All Co_0.5_Mg_x_Cu_0.5-x_Fe_2_O_4_ samples had S-type M-H loops at room temperature, which indicates that the samples are ferrimagnetic. Figure 9 depicts the M-H (magnetic-hysteresis) loops of all samples. The magnetic behavior of the sample changes from soft ferrite to hard ferrite as the Mg^2+^ content increases, as shown in the inset of Fig. 10. Magnetic characteristics for spinel ferrites are shown to vary with ferrite sample composition, cation-preferred occupancy, purity, homogeneity, and structure^26^. Different magnetic characteristics such as saturation magnetization (M_s_), remanent magnetization (M_r_), coercivity (H_c_), and the squareness ratio M_r_/M_s_ were obtained from magnetic hysteresis loops are listed in Table 7. At 8 kG of the applied magnetic field, the ferrite nanoparticles’ magnetic hysteresis loops were unable to fully saturate. This feature is commonly observed in spinel ferrite nanoparticles. Caused by It is brought on by the effect of the size of the ultra-fin ferrite particles and the existence of a spin-disordered layer on nanoparticle surfaces, which must saturate under a strong magnetic field.

**Table 7:** Magnetic parameters of the prepared samples.

X	M_s_ (emu/gm)	M_*r*_ (emu/gm)	H_c_ (Gauss)	Squareness ratio	Anisotropy Constant K*10^3^ (erg/Gauss)
0	42.10	2.60	28.54	0.0618	1.25
0.1	37.89	2.43	24.99	0.064	0.987
0.2	37.97	4.79	41.46	0.126	1.64
0.3	28.95	6.66	56.66	0.230	1.71
0.4	34.63	14.36	517.29	0.414	1.87

As the Mg^2+^ ion substitution increases, the saturation magnetization (M_S_) drops from 42 emu/g to 29 emu/g, as shown in Table 7. The reason for this is the implementation of compositional variation tests. When x = 0.4, the M_S_ rises to 34 emu/g. Because of this, we need to think about how the variation in the distribution of cations changes the contribution of magnetic anisotropy (K) and the magnetic interaction in line with Neel’s and Y-K-type magnetism. Ions from magnetic tetrahedral A and octahedral B sites interact with each other in a super-exchange way, according to the ferromagnet theory. This leads to anti-ferromagnetic alignment (M_B_-M_A_). This leads to magnetization.

According to a new study of Co-Cu ferrites, the ions Fe^3+^, Co^2+^, and Cu^2+^tend to take up tetrahedral and octahedral spots in the crystal lattice^27^. The addition of Mg^2+^ ions to the ferrite lattice, instead of Cu^2+^ ions, does not alter the occupation of Fe^3+^ and Co^2+^ ions between A and B sites. As a result, there is a noticeable decline in the B-B interaction, as well as variations in the antiferromagnetic alignment, with no disruption in the A-B interaction.

At the B-site, there is a canting of spin (Y–K type magnetism). The octahedral site (M_B_) magnetization goes down when Mg^2+^ ion (0µB) is switched out for Cu^2+^ ion (‖1.73 µB) at the octahedral site. This makes the overall net magnetization values go down. However, Table 2 ‘s higher crystallite size in the ferrite sample accounts for the discrepancy found for x = 0.4. The nanoparticle surface effect could be another factor contributing to the sample’s higher M_S_value. Due to environmental asymmetry, an uneven environment causes the magnetic moments on nanoparticle surfaces to be non-collinear. Antiferromagnetic exchange interactions between surface spins cause spin canting. These interactions inevitably lead to a decrease in the spin disorder. In addition, the surface effects cause a magnetic dead layer to form and a core-shell structure to appear. This makes the nanoparticle magnetization even lower than it is in larger ferrite samples^28^.

**Figure 11:**
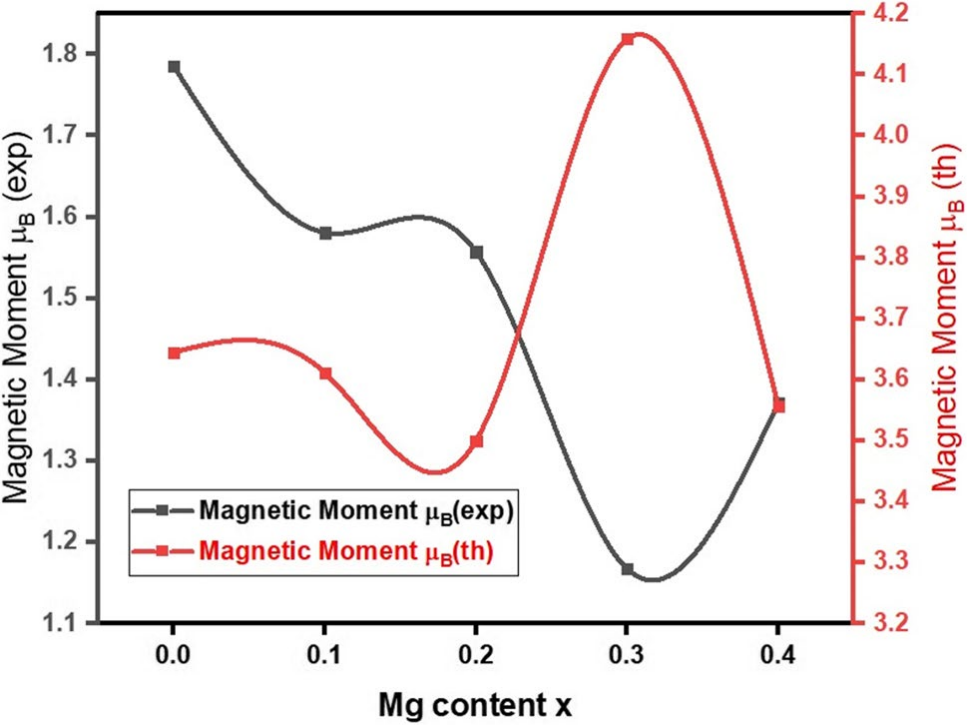
Variation between the theoretical magnetic moment (µ_B(th)_) and the experimental magnetic moment (µ_B(exp)_) with Mg^2+^ content.

Table 7 shows that, up until x = 0.3, the M_S_ values decrease with increasing copper concentration. The exchange contacts and the cation distribution at the A and B sites, respectively, explain the changes in the saturation magnetization. All A-B, A-A, and B-B super-exchange interactions influence ferrite magnetization, although A-B super-exchange interactions are more significant than those of A-A and B-B. Neel’s two-sub-lattice model computes the magnetization of spinel ferrites by assuming that the magnetizations at the B- and A-sites are M_B_ and M_A_, whereas the experimental magnetic moment is determined using the corresponding equations (12), and the values are listed in Table 8 and displayed in Fig. 11.

**Table 8:** Magnetic parameters of the prepared samples.

X	Magnetic Moment µ_B(exp)_	Magnetic Moment µ_B(th)_	Yafet-Kittel angle $\:{\varvec{\alpha\:}}_{\varvec{Y}-\varvec{K}}$	k_1_	H_a_	*N*_d_
0	1.786133913	3.644844	89.23759062	5.31E + 04	2.52E + 03	5.85E + 01
0.1	1.58089514	3.6117	89.26182127	5.25E + 04	2.77E + 03	7.17E + 01
0.2	1.557404391	3.500392	89.25395772	1.04E + 04	5.46E + 02	1.21E + 01
0.3	1.167406857	4.158608	89.37722149	1.47E + 04	1.02E + 03	3.10E + 01
0.4	1.371899092	3.556846	89.28829325	3.65E + 04	2.11E + 03	2.97E + 01

12 $$\:M={M}_{B}-{M}_{A}$$

where M_A_ and M_B_ are the sublattice magnetic moments of the tetrahedral and octahedral sites, M is the molecular weight of the nano ferrite composition.

The first drop in the saturation magnetization value is because the non-magnetic Mg^2+^ ions want to move to the B-sites, which have magnetic moments of 0 µB. This is in contrast to the magnetic Cu^2+^ ions, which want to move to the octahedral (B) site, which has magnetic moments of 1 µB. So, adding Mg^2+^ ions to site B lowers the magnetization of these sublattice points, and Mg^2+^ moves Fe^3+^ from site B to site A. The super-exchange interaction in the ferrite lattice makes the magnetic moment on the B-site stronger. This also makes the magnetic spins of the A and B sites next to each other antiferromagnetically connected. As seen in Fig. 10, the net magnetic moment of the Cu-Mg-Co NPs decreases with Mg^2+^ ion content up to x = 0.3. Multi-magnetic domains are present when R for prepared samples is measured and is smaller than 0.5 (as Eq. (13))^6^.

13 $$\:R={M}_{R}/{M}_{S}$$

The squareness ratio (R), also known as the remanence ratio, is used for identifying whether a material is a single domain or multidomain magnetic, and it was used to provide information about the quality of manufactured nano ferrites. The R values in Table 5are much less than 0.5, indicating the development of single-domain ferrimagnetic particles^9^.

A magnetic material’s magneto-crystalline anisotropy (K) specifies the crystallographic direction that spontaneous magnetization prefers to align within the most energetically advantageous way. It may be calculated using the Stoner-Wohlfarth model whose formula is given in Eq. (14):

14 $$\:K=\frac{{H}_{c\:}\times\:{M}_{s}}{0.96}$$

This model states that H_C_ and M_S_ are necessary for magnetic anisotropy K, and their values range from 5.389 103 erg/g to 0.712 103 erg/g. These numbers fall within the range of ferrimagnetic nanomaterials’ anisotropy constant.

The Yafet-Kittel three-sub-lattice model provides an additional explanation for the variation in magnetic moment. The Yafet-Kittel model divides the B sub-lattice into two sub-lattices, B_1_ and B_2_, each with a triangular spin configuration (canting angle, Y-K). Yafet-Kittel’s model helps to understand the non-collinear or triangular arrangement of spins. We used the following relation^29^ to compute the Yafet-Kittel (Y-K) angles (as Eq. (15)):

15 $$\:{\eta\:}_{B}={M}_{B}\text{cos}{\alpha\:}_{Y-K}-{M}_{A}$$

As Mg^2+^ substitution increases, the α_(Y-K) angles get larger (Table 8). When we add x = 0.4 Mg^2+^, the Y-K angles decrease, indicating an increase in the super-exchange interaction (A-B) and a decrease in the non-collinear arrangement of spins^29^. When α_(Y-K) angles go down, it means that wave functions overlap more with surrounding magnetic ions, which lowers B-B interactions and raises A-B super-exchange interactions^30,31^.

Table 7 lists the coercivity Hc values that were determined. Ferrites’ coercivity is a microstructural characteristic, the table indicates that, except for x = 0.1, the coercivity in the manufactured nano ferrites increases as the amount of Mg^2+^ions increases. The rise could be attributable to the spinel lattice’s surface distortions and crystal flaws^32^. The drop for x = 0.1 may be brought on by the amorphous layer growing on the particle surface, small particles aggregating, or crystallites growing to produce larger particles^21^. Furthermore, several variables, including crystallite sizes, grain sizes, porosity values, morphologies, size distribution, anisotropy constant, saturation magnetization, and magnetic domain affect the coercivity of ferrite^33^. Since the larger particle size distribution lessens the impact of the nanoparticles’ surface area, the higher H_C_ and M_r_values seen for Mg-containing ferrite may be explained by this fact^34^.

At the octahedral sites of the cubic spinel structure, the cobalt ions are known to exhibit substantial anisotropy. Moreover, the Retviled analysis of the samples revealed that Co^2+^ions occupied a sizable portion of A-sites^35^. Co^2+^ions occupy an increasing number of A-sites, leading to low magnetic anisotropy values. Consequently, the environment surrounding the A-sites becomes less anisotropic, leading to a decrease in anisotropy, as noted by Deepak and his colleagues^36^. Table 7illustrates how the magnetic anisotropy increases with the Mg content. Cobalt has a greater LS coupling due to its significant orbital angular momentum, and eventually Mg ions occupy the octahedral sites. The anisotropy in the x = 0.4 sample may be due to the abrupt rise in coercivity, which the increased size of the single-domain NPs causes. Research has demonstrated that the size of the crystallite increases Hc in single-domain NPs^37,38^.

Different types of magnetic materials have been discovered to report the law of approach to saturation magnetization (LAS) in various ways, which is near the saturation (*M*s). According to references^39,40^. In this instance, it was discovered that the law of approach to saturation magnetization is expressed by the following Eq. (16):

16 $$\:M={M}_{s}\left(1-\frac{A}{H}-\frac{B}{{H}^{2}}\right)+\chi\:H$$

Where M_S_ is the spontaneous saturation magnetization of the domains, (H) is the magnetic field values in the region near saturation, the term ($\:\chi\:H$) is the field-induced forced magnetization term, (A) is a constant related to the effects of inclusions and micro-stress, and (B) is a constant related to the contribution of magneto-crystalline anisotropy.

Was found to be appropriate for all the experimental data. In the high field regime (H≫Hc), the magnetization of a material fluctuates with the applied field according to the law of approach to saturation. So, by using LAS (fitting the experimental data with LAS), it is possible to estimate the anisotropy constant and derive details about the mechanisms involved in magnetization. Equation (17) was used to get the anisotropy constant where H_a_ is the anisotropy field.

17 $$\:\begin{array}{c}\varvec{B}=\frac{4{\varvec{K}}_{1}^{2}}{15{\varvec{M}}_{\varvec{s}}^{2}}\\\:{\varvec{H}}_{\varvec{a}}=\frac{2{\varvec{K}}_{1}}{{\varvec{M}}_{\varvec{s}}}\end{array}$$

The anisotropy field H_a_, the first anisotropy constant K_1_, and the saturation magnetization M_s_ can be determined from the straight line of the magnetization M and (1/H^2^) plot in the near saturation field range as reported previously in literature if and only if this plot gives a very well perfect fit indicating that the contributions of the inclusions/micro-stress (A/H) and forced magnetization ($\:\chi\:H$) terms in Eq. (16) are negligible.

## Conclusion

Wet-chemical co-precipitation was used to prepare nano-ferrite samples of Co_0.5_Mg_x_Cu_0.5-x_Fe_2_O_4_ with grain sizes between 8.6 and 15.5 nm. XRD, FT-IR, and EDX analyses confirmed that all samples had a single-phase spinel cubic crystal structure. The lattice constant was increased from 8.3708 to 8.4567 Å. A possible cation distribution shows that Mg^2+^ ions are spread out in both tetrahedral (A) and octahedral (B) sites at the same time but in different amounts. The Fe^3+^ ions make up for the Mg^2+^ ratio deficit. The FTIR spectra of Co_0.5_ Mg_x_Cu_0.5-x_Fe_2_O_4_ samples depict two prominent vibrational mode frequencies that are characterized by tetrahedral and octahedral components in spinel ferrites. Divalent metallic bonds (Mg^2+^-O^2-^, Cu^2+^-O^2-^, and/or Fe^2+^-O^2-^) in octahedral sites may be what cause the absorption band $\:\nu\:$_3_. The force constant of the tetrahedral site (F_A_) is found to be bigger than that of the octahedral site (F_B_), representing stronger covalent bonds in the tetrahedral site. θ_D_ goes down as Mg^2+^ content goes up until x = 0.2. This encourages lattice vibrations, and the samples’ conduction electrons can soak up some of the heat. On the other hand, θ_D_ might go up because there are fewer conduction electrons and more p-type conduction holes. The qualitative chemical composition of the samples shows the presence of all the constituents’ chemical elements (Mg, Co, Cu, Fe, and O) in the final product of Co_0.5_Mg_x_Cu_0.5-x_Fe_2_O_4_ nano-ferrite without any other element and confirms the atomic composition of the material as agreeing with the desired stoichiometry from synthesis. All samples had S-type M-H loops at room temperature, which indicates that the samples are ferromagnetic. The magnetic behavior of the sample changes from soft ferrite to hard ferrite as the Mg^2+^ content increases. M_S_ exhibits an odd behavior, first decreasing from 42.1 emu/g to 28.9 emu/g for x = 0.3, and lastly increasing to 34.6 emu/g for x = 0.4. This may be assigned to the reduction of crystallite size R to a single domain size. In the substitution process, nonmagnetic magnesium (0 µB) replaces the magnetic copper. This weakens the A-B superexchange interactions and makes the spin cant more because of the surface effect. The coercivity of the ferrites goes up as the amount of Mg^2+^ ions goes up. This is what causes the spinel lattice’s surface distortions and crystal flaws.

## Data Availability

All datasets presented in this study are included in the article and can be provided by the corresponding author upon reasonable request.
